# Holistic Care for People Living With Chronic Musculoskeletal Pain: The Relevance and Importance of Sexual Function

**DOI:** 10.1093/ptj/pzae083

**Published:** 2024-07-16

**Authors:** Ilana N Ackerman, Laura Restoux, Brooke Dobo, Helen Slater, Megan H Ross, Andrew M Briggs

**Affiliations:** School of Public Health and Preventive Medicine, Monash University, Melbourne, Victoria, Australia; Physiotherapy Department, Central Coast Local Health District, Central Coast, New South Wales, Australia; Vera Women’s Wellness, Mount Samson, Queensland, Australia; The Wesley Hospital, Brisbane, Queensland, Australia; Curtin School of Allied Health and Curtin enAble Institute, Faculty of Health Sciences, Curtin University, Perth, Western Australia, Australia; School of Health and Rehabilitation Sciences, The University of Queensland, Brisbane, Queensland, Australia; Curtin School of Allied Health and Curtin enAble Institute, Faculty of Health Sciences, Curtin University, Perth, Western Australia, Australia

**Keywords:** Clinical Assessment, Intimate Relationships, Musculoskeletal Pain, Physical Therapy, Referral Pathways, Sexual Function

## Abstract

People living with chronic primary or secondary musculoskeletal pain conditions such as low back pain, fibromyalgia, and inflammatory arthritis typically experience wide-ranging impacts on their physical function, activity participation, and psychosocial wellbeing. These can extend to negative impacts on a person’s sexual function and their intimate relationships. While sexual function is an important component of wellbeing, it is often not considered within musculoskeletal pain care. Without awareness or targeted training, physical therapists may lack the confidence and skills to screen, assess, and manage the impacts that pain may be having on a person’s sexual function and can miss the opportunity to tailor their care and optimize wellbeing. This article seeks to raise awareness among physical therapists of how living with chronic musculoskeletal pain can impact a person’s sexual function and intimate relationships, and provide guidance on how to consider these issues within a person-centered approach to care. It describes why considering sexual function and intimate relationship issues as part of a person’s lived musculoskeletal pain experience may be relevant, outlines the use of validated patient-reported outcome measures to assess sexual dysfunction, and suggests practical strategies for sensitively raising sexual function in consultations. Management approaches and possible referral pathways are also presented, to assist physical therapists in understanding available care options. This article seeks to support holistic care by improving physical therapists’ knowledge and understanding of sexual dysfunction and its management in people living with chronic musculoskeletal pain.

**Impact:**

Considering sexual function as a valued functional activity, together with other activities of daily living, will assist physical therapists to provide more holistic and person-centered care. This article covers the main considerations for raising sexual function and intimate relationship issues with people living with chronic musculoskeletal pain, as well as management options and potential referral pathways. Physical therapists are encouraged to seek targeted training to improve their confidence and skills in this area, and to use inclusive, respectful language for discussions around sexual function and intimate relationships.

## Introduction

In this article, we aim to inform readers about the prevalence and impact of sexual dysfunction (difficulty or impairment in undertaking sexual activity or in experiencing sexual pleasure and satisfaction) experienced by people living with chronic musculoskeletal pain. Incorporating this important, yet potentially overlooked, aspect of a person’s wellbeing into clinical care (when indicated and when in scope of practice) is fundamental to holistic care. We offer practical suggestions for sensitively raising sexual wellbeing in clinical consultations. An overview of assessment and management strategies and potential referral pathways is also provided. We use inclusive definitions of sex, gender identity, sex characteristics, and sexual orientation and intend for this information to be inclusive and broadly applicable across diverse groups.

Why should we consider sexual function among people who seek care for chronic musculoskeletal pain? The importance of identifying and responding to the unique profile of biological, psychological, and social factors that contribute to a person’s experience of chronic musculoskeletal pain is well accepted,^1^ yet sexual function (defined by the American Sexual Health Association as the ability to experience sexual pleasure and satisfaction when desired^2^) may not be explicitly considered. Sexual activity (activity that results in sexual satisfaction and/or meets relational or reproductive needs^3^) is indisputably an important activity for many people, contributing to general wellbeing and healthy relationships.^4–7^ However, sexual function is not usually included in pre-licensure training or routinely considered within musculoskeletal clinical assessment; it is commonly considered the remit of physical therapists who specialize in pelvic health (or related titling, which varies by jurisdiction). The World Health Organization (WHO) identifies sexual health as “fundamental to the overall health and wellbeing of individuals, couples and families”.^8^ We now know, from numerous studies conducted internationally, that sexual dysfunction is relatively common among people who experience chronic musculoskeletal pain. With respect to the WHO International Classification of Functioning, Disability and Health (ICF), sexual dysfunction can be considered across multiple ICF domains including body functions (specifically, genitourinary and reproductive functions, and neuromusculoskeletal and movement-related functions), activity and participation (specifically, interpersonal interactions and relationships), and body structures (specifically, structures related to the genitourinary and reproductive systems).^9^ The burden of sexual dysfunction has been highlighted by recent systematic reviews^10–13^ that have focused on people with inflammatory arthritis (including rheumatoid arthritis, ankylosing spondylitis, systemic sclerosis, and systemic lupus erythematous) and people with chronic non-inflammatory musculoskeletal pain (including fibromyalgia/chronic widespread pain, low back pain, and osteoarthritis). While comparison with general population rates is difficult due to inconsistent definitions and assessment tools, these reviews highlight the high prevalence of sexual dysfunction among women and men, as indicated by low Female Sexual Function Index (FSFI) scores and low International Index for Erectile Function (IIEF) scores, respectively. Additionally, 2 systematic reviews focusing on hip replacement surgery (most commonly performed for osteoarthritis) have shown that sexual dysfunction is relatively common prior to surgery.^14^^,^^15^ On an average, 77% of patients (range 38%–90%) reported “sexual difficulties” prior to surgery and these difficulties started, on average, 2.5 years before hip replacement.^15^

## The Impacts of Chronic Musculoskeletal Pain on Sexual Function

Given consistent associations between chronic musculoskeletal pain and sexual dysfunction or disrupted intimate relationships, there is a need to raise clinicians’ awareness of the impacts of chronic musculoskeletal pain which extend beyond pain, joint swelling, stiffness, and fatigue. In describing these impacts, we recognize the limitations of the current literature, which focuses largely on binary constructs of sex and gender and heterosexual relationships (usually among people who are married), and acknowledge the significant evidence gaps for sexual- and gender-diverse populations who identify as lesbian, gay, bisexual, transgender, queer, intersex, asexual and other related identities (LGBTQIA+). We have also identified in our own systematic reviews^10^^,^^12^ that most research in this area has been conducted on women, suggesting likely knowledge gaps around men’s experiences, which are likely to be different.^12^ Notwithstanding these limitations, there is now substantial evidence that living with painful musculoskeletal conditions can affect diverse constructs related to sexual function, including the initiation (or avoidance) of foreplay and intercourse, sexual satisfaction, sexual desire, sexual distress, self-identity and relationship quality.^10^^,^^12^ Importantly, these impacts have been observed irrespective of pain classification or diagnosis. A meta-analysis of 6 studies involving women with fibromyalgia and sexual dysfunction reported impairments in desire, arousal, orgasm, pain, lubrication, and satisfaction.^16^ A meta-synthesis of 6 studies involving people with inflammatory arthritis conditions found that sexual function was impacted by pain, lower sexual desire, erectile dysfunction, fatigue, fluctuations in disease activity, altered self-image, and confidence in sexuality, with negative impacts on intimate relationships with partners.^10^ Similarly, a meta-synthesis involving people with non-inflammatory chronic musculoskeletal pain found that the experience of pain not only impacted sexual activity, but also intimate relationships, sexual identity, body image, and perceptions of self-worth.^12^ A recent study involving men with inflammatory arthritis confirmed the breadth of sexual function and relationship impacts (based on 34 opinion statements) and emphasized that these impacts vary depending on life stage.^17^

Sexual dysfunction has a multifactorial etiology. While chronic musculoskeletal pain may be an important contributor to a person’s experience of sexual dysfunction, other factors can be involved. These can include trauma, comorbidities or multimorbidity, and interactions between conditions and/or their treatments. Examples include anxiety and depression, endocrine conditions, cancer, neurological conditions, cardiac conditions, chronic pelvic pain, pelvic organ prolapse, urological conditions, colorectal conditions, and gastroenterological conditions,^18^ many of which co-exist with chronic musculoskeletal pain.^19^^,^^20^ Hormonal and physical changes associated with perimenopause and menopause may also play a role, as can alcohol consumption, smoking, illicit drug use and sedentary lifestyles.^21^ Prescribed medications (including serotonin reuptake inhibitors used to treat depression) can also negatively impact sexual function and desire by decreasing pleasurable sensations and delaying or inhibiting orgasm.^22^

## Current Clinical Guidelines for Chronic Musculoskeletal Pain

Contemporary clinical practice guidelines consistently recommend a person-centered, biopsychosocial approach to care, underpinned by shared decision making.^23^ These care recommendations are consistent across clinical guidelines for common chronic musculoskeletal conditions,^24–38^ across reviews of primary guidelines for musculoskeletal pain,^23^ low back pain,^23^^,^^39^ and osteoarthritis,^40^ and in priorities expressed by people living with chronic pain.^41^ While guidelines recommend the holistic assessment of chronic musculoskeletal pain in line with a person’s preferences and priorities,^41^ specific guidance around assessing sexual function and intimate relationships is rarely provided outside of chronic pelvic pain guidelines.^42^ We reviewed widely-used (English language) musculoskeletal clinical practice guidelines and published guideline reviews^23–28^^,^^30–40^^,^^43^^,^^44^ (excluding guidelines for chronic pelvic pain) and identified only 2 guidelines that explicitly recommended that “sexual relationships be considered”^27^ and “sexual advice”^44^ be provided. These both focused on rheumatoid arthritis (RA), which may reflect the known impacts of RA on sexual relationships, due to disease manifestations and the potentially harmful effects of disease-modifying medications in pregnancy. The NICE guideline for the assessment and management of chronic pain^28^ recommends that “social interactions and relationships” be considered, although sexual function is not explicitly mentioned.

## Addressing Sexual Function Within Clinical Consultations

Although studies indicate that many clinicians believe sexual dysfunction should be addressed in health care, it is not routinely included in undergraduate training programs or addressed in clinical practice.^17^^,^^45–54^ A systematic review revealed common barriers to clinicians discussing sexuality with their patients, including clinician discomfort, concerns about causing offense and a perceived lack of training and resources,^48^ which we have highlighted previously.^55^ Some evidence suggests that discussions around sexual function are dependent on a clinician’s age, years of experience, area of practice, comfort with their own sexuality, and level of rapport with their patients.^45^ Similarly, many people do not feel comfortable raising this topic with their treating clinician^55–57^ (even where there is a belief that sexual dysfunction is related to chronic musculoskeletal pain or its pharmacological management^57^) and would prefer clinicians raise sexual dysfunction issues with them.^51^

## Identifying Sexual Dysfunction: The Role of Patient-Reported Measures

Patient-reported outcome measures (PROMs) may be an appropriate starting point for identifying sexual dysfunction as part of holistic care. A range of validated PROMs are available for use; the content, scoring and interpretation of some commonly used instruments are outlined in the Table. In addition to a clinical history, these instruments can provide a snapshot of a person’s current sexual functioning and related impairments, either as a separate construct, or within the broader context of physical functioning and quality of life. Targeted instruments may be preferable in some clinical situations (eg, to assess conditions with pelvic organ or nerve involvement), although this is beyond the scope of this paper. We recognize the lack of validated sexual function evaluation tools for transgender and gender-diverse communities, and that available tools may not include activities relevant to all sexual orientations; this is a barrier to proper assessment and understanding of sexual function.

**Table:** Examples of Relevant Patient-Reported Outcome Measures for Screening and Assessing Sexual Function*^a^*

**Instrument**	**Items**	**Constructs**	**Score Range**	**Available Example**	**Published Clinical Thresholds**
Female Sexual Function Index^74^	19	Sexual desire, sexual arousal, lubrication, orgasm, satisfaction, and pain	2 (worst) - 36 (best)	https://scireproject.com/outcome/female-sexual-function-index-fsfi/	Sexual dysfunction is indicated by a score ≤ 26.5^74^
6-item Female Sexual Function Index^59^	6	Sexual desire, sexual arousal, lubrication, orgasm, satisfaction, and pain	2 (worst) - 30 (best)	https://www.surrey.ac.uk/sites/default/files/2022-09/FSFI-6-information.pdf	Score of <19 indicates a very high probability of sexual dysfunction and need for further investigations^59^
International Index for Erectile Function^60^	15	Erectile function, orgasmic function, sexual desire, intercourse satisfaction, overall sexual satisfaction	Erectile function: 5 (worst) - 30 (best) Orgasmic function: 0 (worst) - 10 (best) Sexual desire: 2 (worst) - 10 (best) Intercourse satisfaction: 0 (worst) - 15 (best) Overall sexual satisfaction: 2 (worst) - 10 (best)	https://www.browardurologycenter.com/pdf/International-Index-of-Erectile-Function-IIEF-Questionnaire.pdf	Erectile dysfunction is indicated by a score ≤ 25 on the erectile function subscale^60^
Changes in Sexual Functioning Questionnaire^75^	14 (CSFQ-14 version, available in female and male versions)	Sexual desire (frequency and interest), arousal/excitement, orgasm/completion	0 (worst) - 70 (best)	https://www.dbsalliance.org/wp-content/uploads/2019/02/Restoring_Intimacy_CSFQ_Handout.pdf	Sexual dysfunction is indicated by a score < 41 for males and a score < 47 for females^75^
WHOQOL-Bref^76^	26 (including 1 item on sex life)	Multi-dimensional (quality of life, health, pain, satisfaction)	Four domain scores each ranging from 4 (worst) - 20 (best)	https://www.who.int/tools/whoqol/whoqol-bref	Not available
Oswestry Disability Index (Oswestry Low Back Pain Disability Questionnaire **-** original version)^62^	10 (including 1 item on sex life)	Pain intensity, personal care, lifting, walking, sitting, standing, sleeping, sex life, social life, and traveling	0% (best) - 100% (worst)	https://www.melbournepaingroup.com.au/sites/default/files/oswestry_disability_scale.pdf	Not available

^a^ CSFQ-14 = Changes in Sexual Functioning Questionnaire-14.

Completion of PROMs instruments that signal potential sexual dysfunction provides clinicians with an opportunity to initiate sensitive conversations. These PROMs could also be used to track improvements or deterioration and to assess the outcome of management strategies. The 2 most widely-published instruments are sex-specific, the FSFI and the IIEF. The FSFI contains 19 items spanning 6 domains that include sexual desire, sexual arousal, lubrication, orgasm, satisfaction, and pain. It is available in more than 20 languages, and a total FSFI score of 26.55 or below is considered to indicate sexual dysfunction.^58^ A shortened 6-item version (the FSFI-6) is also available and covers the same 6 domains as the full-length FSFI; an FSFI-6 score of less than 19 indicates a very high probability of sexual dysfunction.^59^ We note that both versions of the FSFI will produce artificially lower scores if people report “no sexual activity” or that they “did not attempt intercourse” for the relevant items (which could be for reasons other than pain). Therefore, interpretation of FSFI scores should be made with this in mind, and where appropriate, clarified with sensitive communication with the person. The IIEF contains 15 items across 5 subscales. A score of 25 points or less on the erectile dysfunction subscale is considered to indicate erectile dysfunction^60^ (erectile dysfunction is considered a form of male sexual dysfunction^61^). The IIEF has been translated into 32 languages. These instruments are not disease-specific and can be administered regardless of the presenting health condition. Sex-specific versions of the Changes in Sexual Functioning Questionnaire are also available. The non–disease-specific WHOQOL-Bref questionnaire (the short-form WHO quality of life questionnaire) contains 1 item relating to sex life. To our knowledge, the only musculoskeletal disease-specific instrument to consider sexual function is the Oswestry Disability Index (also known as the Oswestry Low Back Pain Disability Questionnaire). The original version of this instrument includes 1 optional item on sex life.^62^ In situations where understanding low back pain-related disability in relation to sexual activity is important, the original version of this instrument is preferable to modified versions that do not include this item. In the Supplemental Material, we provide 2 hypothetical case studies that demonstrate how clinical conversations around low back pain and sexual function might be initiated in different scenarios.

## Sensitively Raising Sexual Function and Impacts on Intimate Relationships

**Figure 1:**
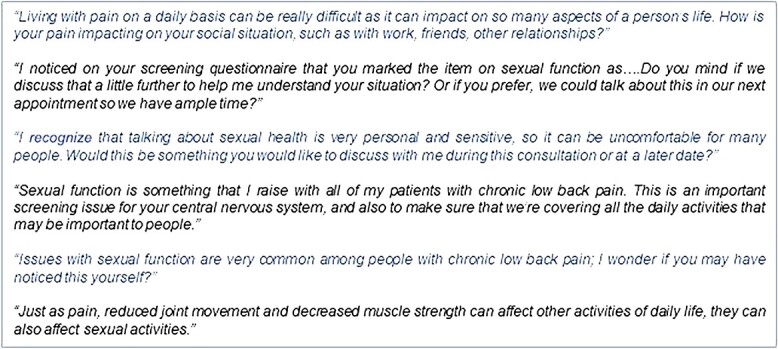
Practical examples for sensitively raising sexual function.

Given a lack of pre-licensure training in this area, clinicians may be understandably reluctant to raise the topic of sexual function with people who experience chronic musculoskeletal pain. We offer the following practice points to assist clinicians in sensitively raising sexual function:

- Ensure that inclusive and appropriate language is used, especially when speaking with people who have diverse gender identities, sexual orientations, or sex characteristics. Inclusive and affirming language is language that ensures people feel seen and respected and avoids assumptions and discrimination. If the person’s preferred terminology around their gender identity is unknown (eg, their preferred pronouns), consider using open-ended questions and allowing them to explain their individual circumstances. Avoid assumptions about sexual activities or behaviors based on gender or sexual orientation.
- Acknowledge it can be difficult for people to communicate their sexual problems to others (Fig. 1) and ask for permission to discuss these issues.^17^^,^^63^ When clinicians take steps to actively listen and legitimize a person’s thoughts and feelings, this provides a safe space to discuss sexual function and intimacy.^64^^,^^65^ Knowing that their experiences are not unusual can be profound, as can the therapeutic benefit of trusted clinical interactions.^64^^,^^65^ A similar approach has been recommended in stroke rehabilitation, where the initial steps of a sexuality interview guide focus on normalizing the presence of sexual difficulties and offering examples of common difficulties.^66^
- Highlight that, as many activities of daily living are affected by chronic musculoskeletal conditions (eg, due to pain, reduced joint mobility, and fatigue), sexual function can also be affected by these issues (Fig. 1).
- Explain that providing holistic care means that sexual function is typically screened as an integral part of a musculoskeletal assessment. Indicate that, together with other functional activities of daily living, this is assessed and reassessed at follow-up, as appropriate and with consent^45^^,^^63^ (Fig. 1).
- Seek explicit consent to include sexual function screening, as appropriate, respecting that in some cultures and contexts (like the presence of past trauma), individuals may not wish to discuss this. Alternatively, people may prefer to return to these discussions at different life stages, for example, when meeting a new partner, planning for pregnancy, or during peri/menopause.
- Maintain professional communication standards. Use respectful, open, and supportive language within a private consultation space to support people to freely discuss their concerns. While humor may help the clinician feel more comfortable, this approach may be inappropriate or not well received.^45^

Other considerations include the need for a safe space for discussions, ideally a private room (rather than cubicles) where the consultation cannot be overheard by others and potential interruptions can be avoided. The involvement of a different clinician may be appropriate, if the person’s preference is to speak with someone of a specific gender. It is important to allow enough time so the person does not feel rushed. To facilitate this, scheduling a specific appointment for these discussions may be preferable, rather than trying to cover too much in a single consultation. Having additional resources available, such as information leaflets or links to online resources, can support conversations and allow people to privately reflect on relevant information.

To support physical therapists in being better equipped and confident to raise these issues clinically, targeted education in sexual dysfunction screening, assessment, and person-focused communication that is sensitive, respectful, and culturally safe is recommended.^17^^,^^45^ This type of education could be provided as part of professional, postgraduate, and continuing education programs, to ensure access for both new and experienced physical therapists. Appropriate education can also assist clinicians to identify and address any beliefs or biases they may hold about sexuality (including biases based on age, gender, culture, or ethnicity),^48^ which could inadvertently contribute to inequities in care. Time and practice in learning effective ways to screen and discuss sexual function with people can enhance clinician confidence^45^ and, coupled with appropriate training, can improve clinician knowledge and expertise in identifying, on-referring, or helping support people with sexual health issues.^50–53^^,^^67–69^ However, educational offerings may not adequately consider diverse populations including people of older age, people who identify as LGBTQIA+ and/or people with specific needs (eg, people with cognitive impairments, physical disabilities, and/or intellectual disabilities),^50^^,^^51^^,^^67^ and further guidance can be sought from clinical colleagues with specific expertise and from relevant consumer organizations.

## Approaches to Person-Centered Management

This section provides physical therapists with an overview of potential person-centered management for sexual dysfunction. Some care will be within scope of practice, whereas other care will require onward referral. Our case studies illustrate how this care may be clinically operationalized (Suppl. Material). First, it is important to identify and understand the person’s experience of sexual function in the context of their musculoskeletal pain, including the contribution of physical and biological factors (musculoskeletal function), psychological factors (general wellbeing, fear, mood, distress, fatigue, trauma), and social context (relationships and social roles). Understanding the person’s preferences and care priorities, including their current and past experiences of care, helps inform a tailored, supportive management plan. Where symptoms of neurological involvement are described (eg as part a spinal assessment), a neurological examination may be indicated. Assessing musculoskeletal function more broadly is relevant, including functional activities, joint range of motion (eg hip and lumbopelvic), strength and conditioning, and identifying unhelpful protective behaviors (hypervigilance, fear, and avoidance).

Physical therapists can also use their expertise to offer advice, which may include education regarding more comfortable sexual positions. Published guidance on safe sexual positions after hip replacement surgery (to reduce the risk of postoperative hip dislocation) is not routinely provided,^70^ but is available from professional body or health services websites.^71^^,^^72^ Advice on activity pacing may also be helpful in the context of pain fluctuations or disease flares, and fatigue that might be most problematic later in the day.^10^

## Referral Pathways

**Figure 2:**
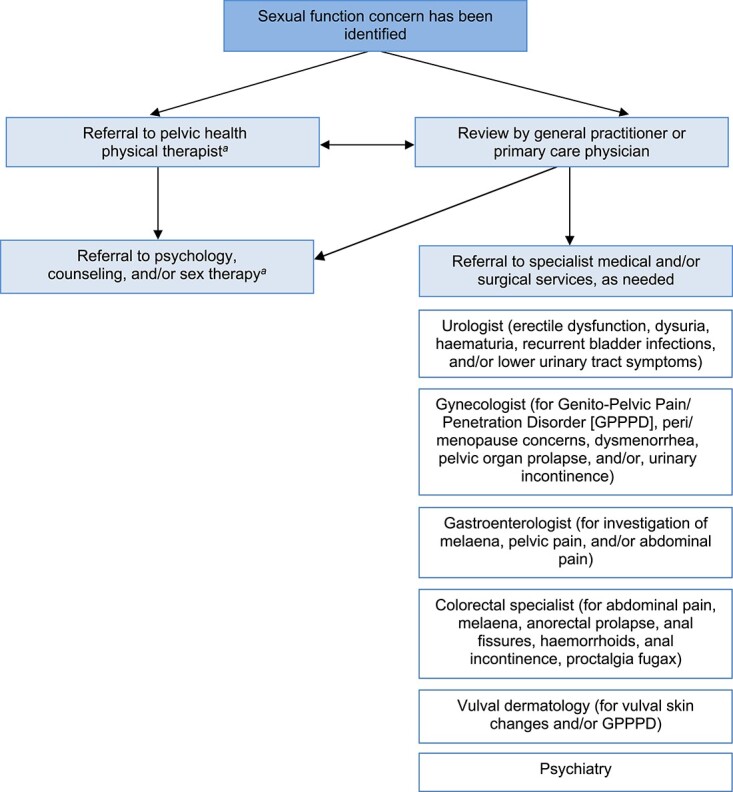
Overview of potential referral options. *^a^* In some jurisdictions, allied health services may require a medical referral.

Individuals with suspected or actual sexual dysfunction (identified eg, during the clinical conversation or through the use of a PROMs instrument) should be referred to an appropriately trained and experienced clinician. Different referral options may be appropriate, depending on the person’s specific needs, priorities, and preferences. A summary of common referral pathways is provided in Fig. 2. It is often appropriate in the first instance to recommend consultation with a pelvic health physical therapist.^73^ If pelvic floor muscle dysfunction is suspected, examination by a pelvic health physical therapist may be indicated (eg a vaginal or rectal examination or transperineal or transabdominal ultrasound) to assess pain, pelvic floor resting tone, muscle coordination, and urogenital tissue integrity. Care provided by pelvic health physical therapists can include specific pelvic floor muscle training (including using biofeedback), manual therapy, muscle relaxation with vaginal or rectal dilator therapy and/or breath awareness, and education around the sexual response cycle and optimizing urogenital tissue health. Potential contributing factors such as signs and symptoms of pelvic organ prolapse, bowel or bladder dysfunction, erectile dysfunction, sexual organ pain, or a history of childhood trauma or sexual trauma may be identified by pelvic health physical therapists and require onward referral.

Further referral options for the comprehensive assessment and management of sexual dysfunction are varied. For some individuals, medical review may be needed. A general practitioner or primary care physician can evaluate whether current medications or comorbidities are contributing to sexual dysfunction. General practitioners and primary care physicians can also provide referrals for specialist medical services and can co-ordinate multidisciplinary care. While referral to medical specialists may not be within the scope of physical therapist practice (depending on the jurisdiction, qualifications, and scope of practice), within a multidisciplinary team-based paradigm it is important for physical therapists to still be cognizant of the available options. Review by a relevant medical specialist (eg rheumatologist, sport and exercise physician, pain physician and/or orthopaedic surgeon) may be needed if musculoskeletal pain factors require additional management. Urology, andrology, or gynecology review may be necessary if the person is experiencing dysuria (pain or discomfort with urination) or dysmenorrhea (painful menstrual periods). Gynecology review may also be warranted for complaints of dyspareunia (painful penetrative intercourse). Referral to a vulval dermatologist may also be appropriate for vulvodynia (pain or discomfort in the vulval area) that may result from psoriasis, lupus, lichen sclerosus, or low estrogen levels in peri/menopausal women. Referral to a gastroenterologist or colorectal specialist may be considered if a person describes abdominal or pelvic pain that requires investigation. Referral to a colorectal specialist may be beneficial for people with anorectal prolapse, chronic anal fissures, or hemorrhoids.

Psychology, psychiatry, relationship counseling, and sex therapy can play an important role in managing sexual dysfunction and a range of medical, cognitive, emotional, and behavioral interventions may be used. These therapies can be helpful in supporting the person in their relationships, addressing lack of desire or arousal, improving sexual satisfaction or orgasm, and addressing previous trauma. Accessing an appropriately trained clinician to help support couples to discuss sexual dysfunction issues may also be appropriate. In some settings, referrals to these types of services may not be required (other than for psychiatry, as a medical specialty) although care coordination by a general practitioner or primary care physician may still be beneficial.

## Conclusion

Sexual function is an important contributor to quality of life and an important activity for many people. Chronic musculoskeletal pain is commonly associated with sexual dysfunction, although significant evidence gaps remain for LGBTQIA+ populations. Ensuring clinicians feel confident to sensitively raise sexual function, as appropriate, is a key starting point given the major role that physical therapists play in musculoskeletal management. An understanding of available screening tools, management approaches, and referral pathways for sexual dysfunction will assist clinicians to provide more holistic, tailored care to people with chronic musculoskeletal pain.

## Supplementary Material

2023-0727_R1_Supplemental_material_Physical_Therapy_pzae083
