# Supplementary material for: Holistic Care for People Living With Chronic Musculoskeletal Pain: The Relevance and Importance of Sexual Function
Source: Phys Ther. 2024 Jul 16;104(8):pzae083. doi: 10.1093/ptj/pzae083 (PMC11367674; doi:10.1093/ptj/pzae083)
Supplement: 2023-0727_R1_Supplemental_material_Physical_Therapy_pzae083 [file 2023-0727_r1_supplemental_material_physical_therapy_pzae083.pdf]

Holistic care for people living with chronic musculoskeletal pain: The relevance and importance of sexual function

Supplemental Material

These hypothetical case studies represents characteristics and contexts that are commonly encountered in musculoskeletal care. The case studies are focused on initial clinical assessment and management within scope of practice for practitioners who are not specifically trained or titled in Pelvic Health physical therapy.

Case study 1

Pam is a 65-year-old woman. She works part-time as a cleaner in a local school. She has been married to her husband for 40 years and they have two adult children who no longer live at home. Approximately two years ago, she sustained an injury to her back at work. Pam was able to make a return to work 12 months ago, returning to her pre-injury hours, but remains on lighter duties.

Pam presents to your clinic complaining of an exacerbation of her chronic low back pain. You are familiar with her history, having seen her 12 months earlier for the same issue and supported her return to work. She currently works part-time as a cleaner at the local school, usually working 3.30 pm to 6.30 pm each day. She loves her job.

While she can still work and perform duties at home, she admits that she is frustrated that her back pain persists and that she feels tired all the time. Pam also admits her mood is "going downhill" and that her pain and "other issues" are getting her down. She is not participating in social activities, due to feeling tired, and is not engaged in any regular leisure physical activity.

You explain to Pam that using a screening tool to quantify her function is important for her care planning and monitoring. Pam agrees to complete the Oswestry Disability Index, scoring 32%. Reviewing the individual items, you notice that she scored item 8 (Sex life) as 4 ('*My sex life is nearly absent because of pain*'). You mention this observation to Pam and ask whether she would be comfortable to discuss this topic further as part of your information gathering, to inform her care planning. She agrees and explains that in the last two years, her sexual activity has been limited due to her chronic back pain and back and hip stiffness. She also experiences reduced libido and arousal and dyspareunia, which has resulted in a desire mismatch with her husband. This has impacted their intimacy.

You ask Pam about her current priorities. She identifies that she wishes to:

- Get back to enjoying her life more: getting out with friends and family again, and getting back to a ‘normal’ relationship with her husband, including intimacy and sexual activity; and
- Continue her job, and to be better able to manage her back pain flares.

On physical examination, you observe no neurological compromise, reduced functional capacity, and limited active movement in her low back and hips. You notice protective behaviors (breath holding, bracing) with bending forwards and reaching to pick things up. Pam feels breathless after performing eight sit-to-stand movements.

You discuss some management options with Pam and jointly develop a care plan, including:

- Helping Pam make sense of her pain and to better understand what is going on and why, and what care options and referral pathways are available;
- Strategies to improve lumbar spine and hip mobility, and target improved general strength and endurance through activity and exercise;
- Education around effective pain management, including:
    - identification of pain triggers and strategies to manage these and pace activities throughout the day and at work;
    - education around positioning in bed to increase comfort during sexual activity and potential medication use that can be discussed with a physician; and
    - education about the use of pain-relieving modalities prior to sexual activity.
- Strategies to improve sleep quality;
- Referral to a Pelvic Health physical therapist (or specialist physical therapist) for the assessment and management of dyspareunia and pelvic floor health and function; and
- Consider recommendation to see her general practitioner for assessment and medical management of dyspareunia, and onward referral to a gynaecologist and sex therapist, if indicated.

As part of the management plan and to inform further tailoring and monitoring, you ask Pam to complete the Female Sexual Function Index and the Depression, Anxiety and Stress Scale (DASS-21) prior to her next consultation to screen for sexual dysfunction and depression, anxiety and stress, respectively. You reinforce that the consultation is a private and safe space and that these discussions can pause or stop at any time. You explain that you will ask Pam to repeat some of the questionnaires again to monitor her progress and adjust her care plan appropriately.

Case study 2

Jay is 34 years old and presents to you with persisting low back pain and stiffness. He works full time in a highly stressful corporate job that demands long hours and frequent travel. Three months ago, he was diagnosed with ankylosing spondylitis (AS). His care is currently managed by a rheumatologist and he has commenced a disease-modifying antirheumatic drug.

At the initial consultation, Jay's primary concern is that he is not able to participate in physical activity due to his persisting back pain and restricted mobility. He describes that he's constantly tired, made worse by interrupted sleep due to back pain and difficulty finding a comfortable position. When you ask Jay about what matters most to him, he reports being frustrated that he can't get on with normal activities, like other people his age. You ask Jay to complete the Bath Ankylosing Spondylitis Functional Index (BASFI), a tool to measure function in adults with AS. You notice that Jay scores high on several items. Given this information, you ask Jay to tell you a little more about how he is coping at work and at home. He mentions that he is now in a relatively new relationship and that his "back stuff" is getting in the way. When you ask for some more information about this and his goals for care, Jay mentions he is mostly missing being able to run and doing "other activities" to relieve stress associated with his busy job.

Jay indicates that as well as running, the constant pain and fatigue are preventing him from being able to focus at work. When you ask about Jay's social context, he mentions that he and his partner Morgan haven't been getting along very well, because Jay is more distant and irritable than usual. You mention that it's common for pain to get in the way of relationships and ask if it would be ok if you asked a little more about this. Jay says this is fine, but when discussing his current circumstances, he is dismissive and indicates it's probably just because he can't run at the moment. You acknowledge that this would be frustrating and distressing for Jay. You explain that living with an autoimmune condition like AS and the impacts of constant pain can impact many aspects of

someone's quality of life, from functional limitations at work and home, as well as impacts on relationships, and for many men, their sexual function. Jay agrees that his quality of life is definitely affected.

You ask whether his partner knows much about AS. Jay replies that his partner hasn't attended any appointments with his rheumatologist, so they probably don't understand. You mirror Jay's non-gendered language when talking about his partner.

You explain that, in addition to the BASDI, you would like Jay to complete a screening tool that will help to develop a management plan targeted to Jay's goals and improving his quality of life. Jay agrees to complete the WHOQOL-Bref. In reviewing the individual items, you notice that Jay's mental health and satisfaction domains were scored with high impact, and you identify that he responded with 1 for "*How satisfied are you with your sex life?*". You ask if he would be comfortable discussing this in more detail with you. Jay appears hesitant, so you suggest that perhaps you can talk about it at the next appointment if he would like to.

On physical examination, Jay is limited in his low back range of movement, especially rotation and extension. His hip mobility is limited bilaterally, with associated hip musculature limitations. Together you discuss a management plan to address Jay's goals, including:

- Developing and implementing a spinal mobility plan to address back pain and stiffness at work and at home.
- Starting a gentle and graded aerobic conditioning program to maintain fitness for running and endurance at work. You jointly agree that pool-based activities might be a good place to start.
- Implementing pain management strategies, including relaxation and pacing, to improve pain coping.
- Involving his partner in the management plan.

At the end of the first consultation, you reassure Jay that you can both work together towards his goals and that the consultation is a safe and confidential environment to talk about any issues. You explain to Jay that, in relation to his WHOQOL-Bref scores, it is common for men with AS to experience sexual function impairments and that perhaps formerly enjoyable positions or activities may now feel awkward or uncomfortable, or that he may be too tired to want to have sex. You reassure Jay that while sexual dysfunction is common with AS, there are ways to manage it. You ask Jay, if he feels comfortable, to complete the International Index of Erectile Function (IIEF) at home and bring it to the next appointment, and perhaps invite his partner to attend. Jay agrees, and you email the questionnaire for him to complete.

You explain to Jay that you can use the scores of the BASFI, WHOQOL-Bref and IIEF questionnaires to monitor his progress over time and adjust his management plan according to his needs. Depending on how he feels over time, you explain that it may be helpful to consider:

- Developing strategies to have sex more comfortably, involving his partner
- Communicating his concerns with his rheumatologist to ensure pharmacological management is optimized
- Accessing support services for his mental health, if needed
- Exploring other referral options (like sex therapy and relationship counseling) to assist with supportive management of his relationship, including sex and intimacy, if he feels this would be of benefit
